# Exploring K_*v*_1.2 Channel Inactivation Through MD Simulations and Network Analysis

**DOI:** 10.3389/fmolb.2021.784276

**Published:** 2021-12-20

**Authors:** Flavio Costa, Carlo Guardiani, Alberto Giacomello

**Affiliations:** Dipartimento di Ingegneria Meccanica e Aerospaziale, Sapienza Università di Roma, Rome, Italy

**Keywords:** Shaker, Kv1.2, C-type inactivation, molecular dynamics, network analysis

## Abstract

The KCNA2 gene encodes the K_*v*_1.2 channel, a mammalian Shaker-like voltage-gated K^+^ channel, whose defections are linked to neuronal deficiency and childhood epilepsy. Despite the important role in the kinetic behavior of the channel, the inactivation remained hereby elusive. Here, we studied the K_*v*_1.2 inactivation *via* a combined simulation/network theoretical approach that revealed two distinct pathways coupling the Voltage Sensor Domain and the Pore Domain to the Selectivity Filter. Additionally, we mutated some residues implicated in these paths and we explained microscopically their function in the inactivation mechanism by computing a contact map. Interestingly, some pathological residues shown to impair the inactivation lay on the paths. In summary, the presented results suggest two pathways as the possible molecular basis of the inactivation mechanism in the K_*v*_1.2 channel. These pathways are consistent with earlier mutational studies and known mutations involved in neuronal channelopathies.

## 1 Introduction

The KCNA2 gene encodes the K_*v*_1.2 channel, a mammalian voltage-gated K^+^ channel featuring up to 80*%* homology with the *Drosophila* Shaker channel (Suárez-Delgado et al., 2020). It is widely expressed in mammals by visceral smooth muscle cells (Wang et al., 1994) and neurons of the central and peripheral nervous system (Niday and Tzingounis, 2018). Its defections/malfunction are linked to neuronal deficiency inducing encephalopathies, ataxia, cerebellar atrophy (Morrison-Levy et al., 2020), and especially childhood epilepsy (Masnada et al., 2017).

**FIGURE 1:**
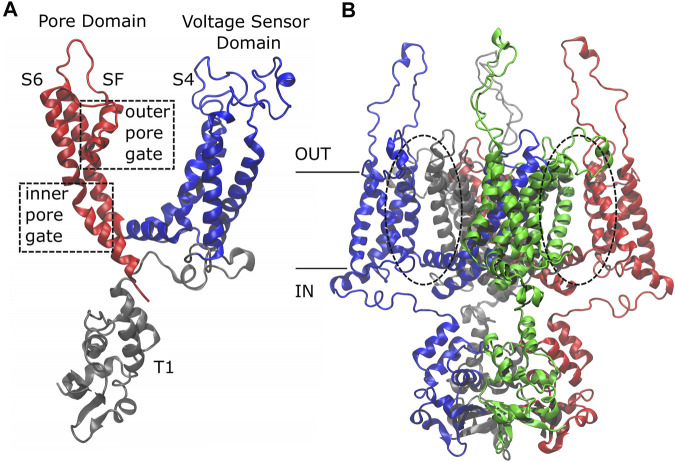
Side view of the K_*v*_1.2 channel. The single subunit **(A)** is colored by domain: in gray, the T1 domain at the N-term; in blue, the Voltage Sensor Domain (VSD) ranging from helix S1 to loop L45, including the positively charged helix S4; in red, the Pore Domain (PD), composed of helix S5, P-Loop, Selectivity Filter, and helix S6. The whole protein **(B)** is colored by subunit: the first subunit in blue, the second in red, the third in gray, and the fourth in green. Note that the color code has a different meaning in the two sub-figures.

Initially, this channel was studied using the structure solved by MacKinnon and colleagues of a modified rat K_*v*_1.2 channel where the voltage sensor paddle, encompassing the S3 and S4 helices, was replaced by the voltage sensor paddle from the rat K_*v*_2.1 channel, the so-called “paddle-chimera channel” (Long et al., 2007). Then, the structure of the K_*v*_1.2 channel was experimentally solved (Chen et al., 2010), revealing that it is characterized by four identical subunits made of six *trans*-membrane alpha helices each. There are three functional domains: the T1 domain at the amino-terminus, the Voltage Sensor Domain (VSD) that encompasses helices S1 to helix L45 and is sensitive to the membrane potential variation triggering the channel to open, and the Pore Domain (PD) delimited by the S5 and S6 alpha helices with the P-Loop and the Selectivity Filter (SF) (Figure 1A).

Based on the protein architecture, the Kv1.2 channel is classified as a domain-swapped channel where the peripheral VSDs interact with the PDs of the *neighboring* subunit (dashed circles in Figure 1B). These channels are characterized by a long helical S4–S5 linker (also referred to as L45) that acts as a mechanical lever pushing onto helix S6 of the neighboring subunit and straightening it to close the pore. These are the main features of the domain-swapped channels canonical activation/deactivation mechanism. On the other hand, in non-domain-swapped channels, the linker L45 is non helical and so short that it is unlikely to exert a force on S6 (Barros et al., 2019).

In most K^+^ channels, the ion permeation is regulated by conformational changes occurring at least at opposite sites of the PD: the bending of the S6 alpha helices (at the bottom of S6) causes the inner gate to close, determining the transition from the Open (O) to the Closed (C) state of the channel (Liu et al., 1997; Doyle et al., 1998; del Camino and Yellen, 2001; Long et al., 2005; Jensen et al., 2012). On the other hand, structural rearrangement of the SF that forms the outer gate is the cause of the transition from the O to the Inactivated (I) state, a slow process defined as C-type inactivation (Hoshi et al., 1991; Baukrowitz and Yellen, 1995; Ader et al., 2009). The I state plays a key role in the kinetic behavior of the channels because it is reached immediately after the inner gate opening and, likewise, it allows the channel to close leading the conformation of the inner gate towards the closed state (Monod et al., 1965; Cuello et al., 2010). Despite its central role in channel function, the molecular determinants of the inactivation remained quite elusive.

The transitions O → I and I → C and *vice versa* are regulated by the membrane potential variation, implying a functional connectivity between the VSD and the SF (Olcese et al., 1997; Loots and Isacoff, 1998). Previous computational studies on Shaker activation/deactivation revealed the existence of an alternative pathway that connected the VSD to the PD excluding the long loop L45 (Fernandez-Marino et al., 2018). Successively, experimental evidences supported the idea of a non-canonical activation/deactivation mechanism in the VSD-PD coupling that involved the S4\S5 and S5\P-Loop interfaces (Carvalho-de Souza and Bezanilla, 2019). Moreover, a very similar non-canonical pathway involving the S1 and S5 helices was identified in hERG, a non-domain-swapped channel, where it contributed to the pore opening (Costa et al., 2021). Finally, Bezanilla and coworkers showed that this alternative pathway operates independent of the canonical coupling endowing the voltage dependence to the SF, suggesting a key role in the inactivation mechanism (Bassetto et al., 2021). In order to characterize the dynamic coupling between VSD and SF in K_*v*_1.2 inactivation, in this work, we studied the motion propagation between these two regions analyzing equilibrium fluctuations of the open state. The rationale of this strategy is that the open state immediately precedes the inactivated state in the functional cycle of K_*v*_1.2 with small conformational differences between the two states (Panyi and Deutsch, 2006); in particular, we used the SF as the final endpoint of our search, which is responsible for C-type inactivation (see below).

Based on the KcsA studies about the influence of the inner pore gate on the conformation of the outer pore gate (Cuello et al., 2010), it has been demonstrated that, in Shaker, the residue I470 at the bottom of S6 helix plays a critical role in the C-type inactivation: when the pore opens, this residue modifies its side-chain orientation and physically makes contact with the SF, inducing a constriction capable of stopping the ion flow (Peters et al., 2013). These experimental evidences support the idea of a different coupling mechanism implicated in the C-type inactivation driven by the modifications of the S6 helices after the channel opening and not by a direct coupling with the VSD. Then, it has been shown that the C-type inactivation in hERG, a non-domain-swapped channel, depends on residue F627 of the SF that blocks rapidly the ion flux (Li et al., 2015); similarly, Shaker-like *K* ^+^ domain-swapped channels are characterized by this type of inactivation (Hoshi et al., 1991; Baukrowitz and Yellen, 1995; Ader et al., 2009). It is notable that this inactivation mechanism completely occurs within the PD so that a mechanism occurring in a non-domain-swapped channel like hERG may also apply to domain-swapped channels of the Shaker family. After the sequence alignment between hERG and Kv1.2 (shown in Supplementary Figure S2), it is evident that in the SF of Kv1.2, only Y377 has a side-chain characterized by a steric hindrance similar to that of the phenylalanine on hERG, which suggests that Y377 may have a similar role to F627 in hERG. For this reason, in addition to VSD-SF couplings, we studied in detail the pathways connecting the PD to the side-chain re-orientation of Y377 that was chosen to be the key residue of the sink region on the SF for the network analysis (for more details, see *Methods*).

The C-type inactivation in the K_*v*_1.2 channel seemingly originates in VSD-SF and PD-SF couplings. Thus, characterizing microscopically this transition amounts to study how conformational changes of the S4 sensor helix and the pore-delimiting S6 helix propagate to the SF. Considering the long-range conformational re-arrangements of the channels occurring during the transitions from one functional state to another, ion channels can be classified as allosteric molecules (Changeux et al., 1984). A promising approach to study allosteric proteins examines how the fluctuations of residue pairs correlate over the course of an equilibrium simulation to reconstruct the propagation of motion across the protein network (Elbahnsi and Delemotte, 2021). This approach was successfully employed to study the long-range communication path in the E2 enzymes (Papaleo et al., 2012) and in the tRNA synthetase (Ghosh and Vishveshwara, 2007). It has recently been applied also to ion channels, in particular to the Kv1.2–2.1 chimera (Fernandez-Marino et al., 2018) and hERG (Costa et al., 2021) to clarify how motion propagation determines their activation and deactivation. Here, we applied this network theoretical approach to clarify the molecular determinants of inactivation in the K_*v*_1.2 channel.

## 2 Methods

The initial configuration of K_*v*_1.2 was taken from the experimentally solved open structure (PDB ID 3LUT) (Chen et al., 2010). Using the CHARMM membrane builder (Jo et al., 2008, 2009), it was embedded in bilayers of 880 1-palmitoyl-2-oleoyl-sn-glycero-3-phosphocholine lipids with 65,408 TIP3P water molecules and 0.15 M of KCl to form a simulation box of ca. 150 × 150 × 175 Å totaling 259,933 atoms. The APBS server (Baker et al., 2001) was used to analyze the protonation states of residues: all aspartates and glutamates were ionized; H271, H378, and H486 were predicted to be in the *δ* protonation state; H264 was assigned to the *ϵ* protonation state. Then, the channel was equilibrated in the NPT ensemble for 100 ns. Mutants were produced from the pre-equilibrated wild-type system. At first, they were equilibrated for 6.5 ns in the NPT ensemble applying a time-varying harmonic restraint on each mutated residue and on its neighbors within a cutoff distance of 5.0 Å. The force constant, initially set to 10 kcal/mol/Å, was decreased by 2 units every 0.5 ns of this simulation and then they were equilibrated for 50 ns in the NPT ensemble. All simulations were run with NAMD (Phillips et al., 2005) using the ff14SB force field for the protein (Maier et al., 2015) and the Lipid17 force field for the lipids (Dickson et al., 2014). Pressure was kept at 1.01325 Bar by the Nosé-Hoover Langevin piston method (Martyna et al., 1994; Feller et al., 1995) and the temperature was maintained at 303.15 K by a Langevin thermostat with damping coefficient of 1 ps^−1^. Long-range electrostatic interactions were evaluated with the smooth Particle Mesh Ewald algorithm with a grid space of 1 Å. For short-range non-bonded interactions, a cutoff of 12 Å with a switching function at 10.0 Å was used. The integration time step was 2 fs.

The contact map analysis was carried out computing for each couple of residues the probability to be in contact during the equilibrium simulations. Precisely, two residues were considered to be in contact when a pair of heavy atoms of the side chains was closer than 5.0 Å for at least 75% of the trajectory.

For the network analysis, the protein was represented as a graph (Bondy and Murty, 1976) where nodes correspond to residues and edges to interactions between pairs. Edge weights were calculated using: $d_{\text{ij}}=\;-\log\left|{Corr}_{\text{ij}}\right|$ where Corr_ij_ is the correlation coefficient, that is, the normalized covariance of C_*α*_ positions:

$${Corr}_{\text{ij}}=\frac{\left\langle \left({\vec{r}}_{i}-\left\langle {\vec{r}}_{i}\right\rangle \right)\left({\vec{r}}_{j}-\left\langle {\vec{r}}_{j}\right\rangle \right)\right\rangle }{\sqrt{\left\langle {\left({\vec{r}}_{i}-\left\langle {\vec{r}}_{i}\right\rangle \right)}^{2}\right\rangle \left\langle {\left({\vec{r}}_{j}-\left\langle {\vec{r}}_{j}\right\rangle \right)}^{2}\right\rangle }}$$ (1)

where ${\vec{r}}_{i}$ and ${\vec{r}}_{j}$ are the position vectors of the *i* and *j* residues and Corr_ij_ assumes values in the interval [−1, 1]. Spheres of radius 6 Å were centered on two key residues on S4 and S6 helices and on SF of the neighboring subunit to identify all residues inside them for at least 75% of the trajectory defining the source and sink regions. Shortest pathways were computed using Dijkstra’s algorithm (Dijkstra et al., 1959). The betweenness of each residue was computed with Brandes algorithm (Brandes, 2001) as implemented in the NetworkX library (Hagberg et al., 2008). For more details, see *Supporting Methods*.

## 3 Results

### 3.1 Network Analysis: The Inactivation Pathways

**FIGURE 2:**
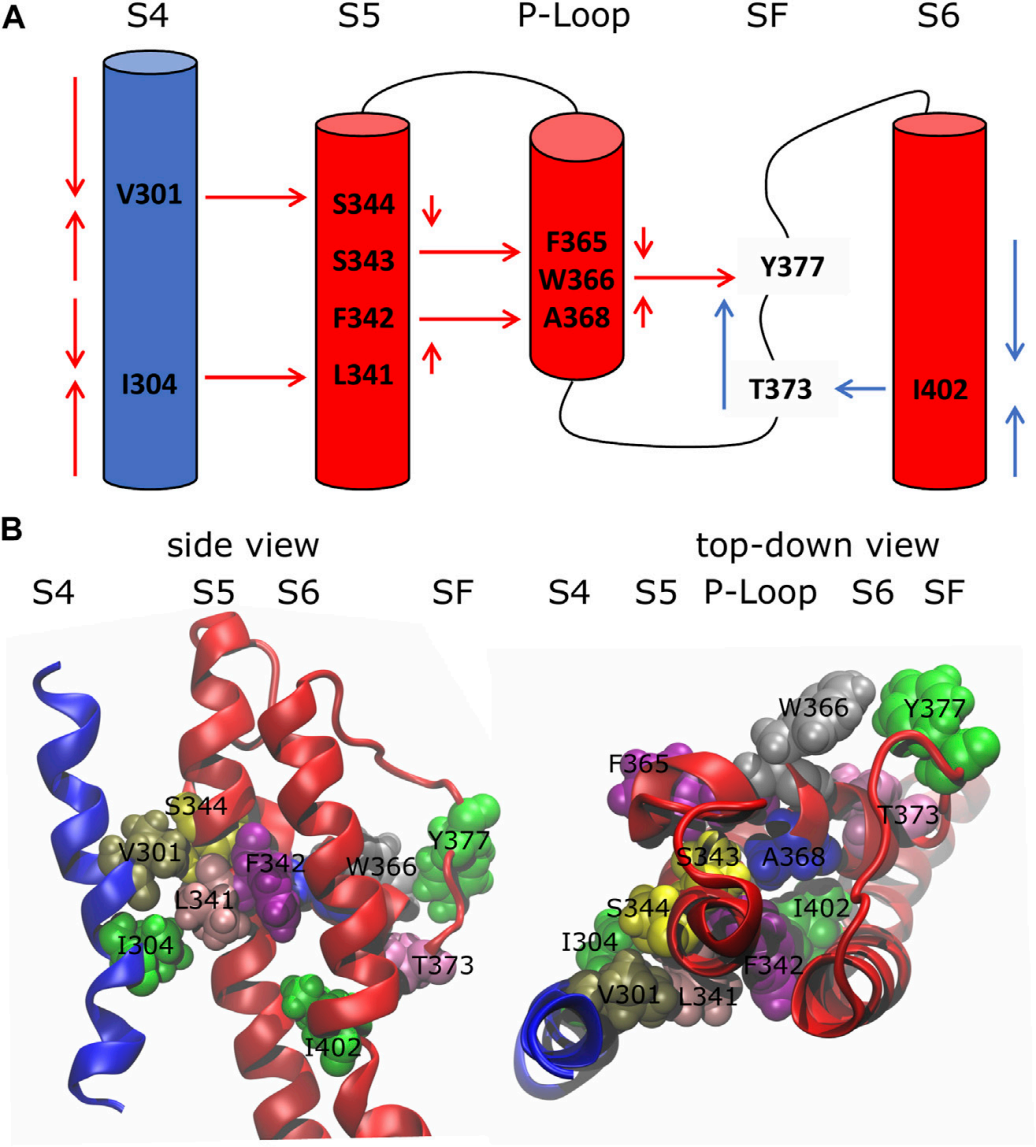
Inactivation pathways of the wild-type K_*v*_1.2 channel. **(A)** Arrows describe the preferred routes of motion propagation: red arrows refer to S4 → S5 → P-Loop → SF route; blue arrows refer to the S6 → SF route. **(B)** Side and top-down views of residues implicated in the paths at the interfaces S4\S5, S5\P-Loop, P-Loop\SF and S6\SF.

Using the network-theoretical approach (see *Methods*), we identified two different families of pathways for the motion propagation (Figure 2) joining the VSD and SF and the PD and the SF, respectively. In the first case, the motion from the top of helix S4 went down and jumped onto helix S5 of the neighboring subunit at the level of the residues V301 and S344; at the same time, from the bottom of helix S4, it went up and passed to helix S5 of the neighboring subunit with I304 and L341. Then, through the P-Loop it reached the SF (red arrows in Figure 2A) where the motion of Y377 could affect the dynamics of its counterpart Y377 on the SF of the subunit where the pathway was originated. The VSD-SF path length averaged for the four subunits was ca. 1. This value is expressed by the sum of the arc lengths (weights) *d*_ij_ that are traversed along a path of minimal length connecting residues *i* and *j*. Arc weights are computed according to equation $d_{\text{ij}}=-\log\left|{Corr}_{\text{ij}}\right|$ where Corr_*ij*_ is the correlation coefficient that measured how efficiently the information was transferred from one residue to the other (see *Methods*). Considering the logarithmic nature of this metric, the length value is a pure number, which is zero only for perfect correlation. Values in the range 0 < *d*_*ij*_ < 2 typically mean that there is a high correlation of the motion of each pair of residues on the path. In the same way, we saw a direct connection between the inner and the outer pore gates (PD-SF coupling) involving the residues I402 and T373 of the same subunit with an average path length of ca. 1.10 (blue arrows in Figure 2A).

The centrality index (CI) and the betweenness (B) of each residue implicated in the paths are shown in Table 1: high values correspond to residues that act as hubs in the communication pathways and thus are expected to play a key role in the motion propagation.

**TABLE 1:** Centrality index (CI) and betweenness of each residue implicated in the paths. Legend Betweenness (B): low 0 < *B* ≤ 1; medium: 1 < *B* ≤ 4; high: 4 < *B* ≤ *B*_max_. Residues whose mutations are implied in epileptic encephalopathy are shown in bold.

Residue	Centrality index (CI)	Betweenness
**L293**	0.10 ≤ *CI* ≤ 0.15	Low
R294	0.10 ≤ *CI* ≤ 0.15	Low
V295	0.10 ≤ *CI* ≤ 0.20	Low
I296	0.20 ≤ *CI* ≤ 0.25	Low
R297	0.30 ≤ *CI* ≤ 0.35	Low
L298	0.30 ≤ *CI* ≤ 0.45	Medium
V299	0.40 ≤ *CI* ≤ 0.55	Medium
R300	0.50 ≤ *CI* ≤ 0.60	Medium
V301	0.70 ≤ *CI* ≤ 0.80	High
**F302**	0.70 ≤ *CI* ≤ 0.80	High
R303	0.70 ≤ *CI* ≤ 0.80	High
I304	0.80 ≤ *CI* ≤ 0.90	High
F305	0.70 ≤ *CI* ≤ 0.85	Medium
K306	0.70 ≤ *CI* ≤ 0.80	Medium
L307	0.60 ≤ *CI* ≤ 0.70	Medium
S308	0.40 ≤ *CI* ≤ 0.55	Medium
R309	0.40 ≤ *CI* ≤ 0.55	Medium
H310	0.10 ≤ *CI* ≤ 0.20	Low
S311	0.10 ≤ *CI* ≤ 0.20	Low
L341	*CI* ≥ 0.90	High
F342	*CI* ≥ 0.90	High
S343	*CI* ≥ 0.90	Medium
S344	0.50 ≤ *CI* ≤ 0.70	Medium
F365	0.75 ≤ *CI* ≤ 0.85	High
W366	*CI* ≥ 0.95	High
W367	0.90 ≤ *CI* ≤ 0.95	High
A368	*CI* ≥ 0.95	High
T373	*CI* ≥ 0.95	High
T374	0.90 ≤ *CI* ≤ 0.95	High
V375	*CI* ≥ 0.95	High
G376	0.80 ≤ *CI* ≤ 0.90	Medium
Y377	*CI* ≥ 0.95	High
C394	0.10 ≤ *CI* ≤ 0.15	Low
A395	0.10 ≤ *CI* ≤ 0.20	Low
I396	0.20 ≤ *CI* ≤ 0.25	Low
A397	0.20 ≤ *CI* ≤ 0.30	Medium
G398	0.30 ≤ *CI* ≤ 0.45	Medium
V399	0.50 ≤ *CI* ≤ 0.60	Medium
L400	0.70 ≤ *CI* ≤ 0.85	Medium
T401	0.80 ≤ *CI* ≤ 0.90	High
I402	*CI* ≥ 0.95	High
A403	0.80 ≤ *CI* ≤ 0.90	High
L404	0.70 ≤ *CI* ≤ 0.80	Medium
**P405**	0.40 ≤ *CI* ≤ 0.50	Medium
V406	0.20 ≤ *CI* ≤ 0.40	Medium
P407	0.20 ≤ *CI* ≤ 0.35	Medium
V408	0.10 ≤ *CI* ≤ 0.20	Low
I409	0.10 ≤ *CI* ≤ 0.20	Low
V410	0.10 ≤ *CI* ≤ 0.20	Low

### 3.2 Contact Map Analysis

**FIGURE 3:**
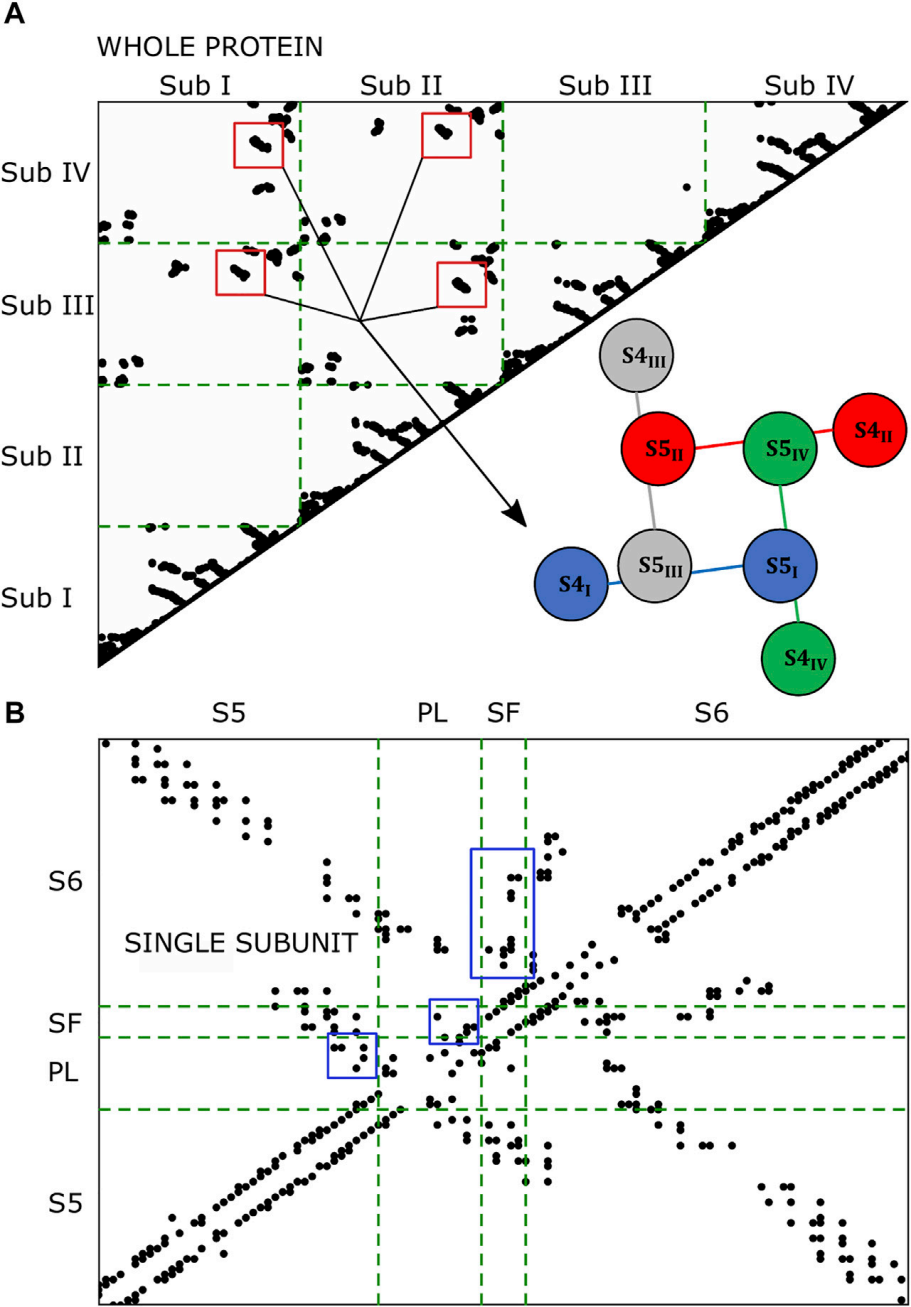
Contact maps of the whole protein **(A)** and of the single subunit **(B)**. Black dots represent the formed interactions. In the single subunit map, the black dots are the interactions formed at least 75% of the trajectory in 3/4 subunits.

The microscopic characterization of the inactivation pathways was done computing a contact map that highlighted all the conserved interactions formed between residues of the same or the neighboring subunits. Figure 3 represents the contact map of the whole protein and of a single subunit where black dots are the formed interactions between residues for at least 75% of the equilibrium trajectory. The contact pattern shown in the whole protein map confirmed the presence of interactions between helices S4 and helices S5 of the neighboring subunit (red box in Figure 3A). Besides, the contacts formed between helices S5 and the P-Loop and helices S6 and the SF of the same subunit (blue box in Figure 3B) supported the second part of the paths that we identified in Figure 2A.

### 3.3 Mutants

The network approach allowed to identify two routes of the motion propagation as the molecular basis of a VSD-SF and PD-SF coupling mechanisms. The residues that connect the interfaces S4\S5, S5\P-Loop, P-Loop\SF, and S6\SF are strongly coupled in the equilibrium dynamics of the open state. Since they lie on paths that reached the SF excluding the L45 known to be involved in the activation/deactivation of the channel (Barros et al., 2019), it can be hypothesized that they are involved in the channel inactivation. Many of them have been already demonstrated to influence the Shaker or K_*v*_1.2 inactivation if mutated including L361R, L366H, and W366F (Perozo et al., 1993; Yang et al., 2007; Cuello et al., 2010; Cordero-Morales et al., 2011; Bassetto et al., 2021) but a microscopic interpretation of the effects of the mutation is still elusive. Moreover, it was hypothesized that the VSD-SF coupling depends on the volume of the residues that lay along the inactivation pathway (Bassetto et al., 2021). For this reason, in order to describe the microscopic role of each residue implicated in the paths, we applied the same network-theoretical approach to mutated channels and then we computed a contact map to identify the effects of the mutations on the contact formation. In some cases, we reproduced computationally experimental mutants whose effect on the inactivation was already characterized in Shaker channels, e.g., L361R, L366H, W434F. In order to further characterize the role of key residues lying along the pathways coupling the VSD and the PD to the SF and for which an experiment was not available, we produced a computational model of K_*v*_1.2 with non-conservative mutations obtained by replacing individual residues by an alanine. All the mutated residues on the sequence of Shaker and Kv1.2 channels are shown in Supplementary Figure S1 and the corresponding paths lengths are shown in Table 2.

**TABLE 2:** Comparison of the average path length between WT and mutants.

Mutation	VSD-SF path length	PD-SF path length
WT	0.97	1.12
L293R	9.79	2.78
L298H	5.44	1.36
V301A	1.72	1.31
I304A	3.01	1.50
F342A	5.61	1.49
S344A	1.58	1.37
W366F	0.62	1.71
T373A	1.06	2.32
I402A	1.37	4.77

At first we focused on the VSD-SF coupling path, starting from residues of the voltage sensor helix S4: L293R, L298H, V301A, I304A. In all cases the coupling paths for the inactivation were qualitatively similar to the wild type but with a different path length. The greatest effects were observed mutating L293 on the top of S4 helix, with the average path length reaching values of ca. 10 in the L293R mutant. It probably depended on the introduction of a new positively charged amino acid that influenced the sensitivity to the membrane potential variation. Considering the logarithmic nature of this metric, a difference of two units in the path length corresponds to a difference of one order of magnitude in terms of correlation, meaning that the pathway is effectively hindered in the mutant. These results are in agreement with the experiments on Shaker where L361R channels activated and inactivated at much more hyperpolarized membrane potentials, implying that the inactivation was not preserved (Yang et al., 2007).

**FIGURE 4:**
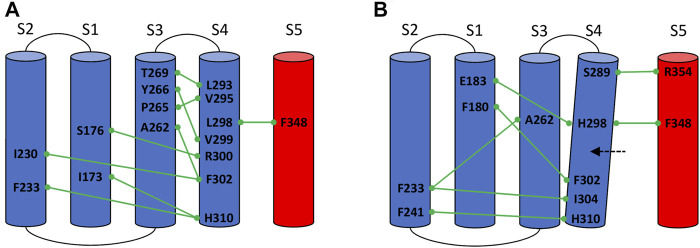
Conserved interactions formed between helix S4 and helices S1, S2, S3, and S5 of the same or neighboring subunits in the wild-type **(A)** and in the mutated L298H channel **(B)**.

In the L298H channel the VSD-SF coupling was extremely weak. The contact map analysis revealed the formation of new contacts between helix S4 and helices S1 and S2 (Figure 4). More precisely, in the mutant, the new histidine maintained the hydrophobic interaction with F348 on helix S5 but its larger steric clash induced a displacement of helix S4 by 3 Å towards helices S1 and S2. We hypothesize that the rearrangements of helix S4 is at the origin of the modification of the inactivation pathways. These computational evidences seem to be in agreement with the experimental Shaker double mutant L366H:W434F where the currents show a decay under sustained voltage-clamp depolarization reminiscent of C-type inactivation, suggesting this process has not been eliminated but rather mitigated. Indeed, L366H relieves the W434F effect of the inactivation increased speed, converting a non-conductive channel in a conductive one (Bassetto et al., 2021).

In the I304A channel the efficiency of the information transfer from VSD to SF became weaker than the wild type. The missing hydrophobic interaction between A304 and L341 on helix S5 is probably the main cause of the loss of inactivation. Interestingly, we identified a new route that from helix S4 reached helix S5 jumping onto helix S1 of the same subunit at the level of R300-F180 contact but this path had a length greater of ca. 3 (see Table 2). Consequently, in I304A channel the inactivation seems to be delayed or completely abolished.

For both V301A and S344A mutants we saw a path qualitatively and quantitatively (length ca. 1) similar to the wild type, meaning that the inactivation would not be affected by the alanine substitutions. Indeed, the contact map analysis revealed that the hydrophobic interactions at the S4\S5 interface were preserved. These results agree with the thesis that the VSD-SF coupling depends on the volume of the residues involved in the inactivation pathway (Bassetto et al., 2021).

Residue F342 on helix S5 plays a central role in the inactivation path connecting the VSD to the SF. Indeed, it is characterized by a very high CI. To further dissect its role in this path, we introduced an alanine substitution in each subunit. The mutated channel F342A had a loss of this coupling mechanism. Interestingly, we saw that the F342–A368 interaction was completely broken by the alanine replacement probably due to the smaller size of the hydrophobic region of the residue.

On the other hand, in the W366F channel, we identified the same VSD-SF path as in the wild type but with a lower average length of ca. 0.60. Here, the efficiency of the information transfer was greater than the wild type, suggesting the presence of an enhanced and faster inactivation in agreement with the experimental results on the K_*v*_1.2 channel (Cordero-Morales et al., 2011) and on the corresponding W434F of Shaker (Perozo et al., 1993). However, the contact map analysis did not reveal new broken or formed interactions.

Finally, we focused on the PD-SF coupling mechanism where we performed the mutations T373A and I402A. In both mutants there was an increase of the path length, meaning that the information transfer was generally less efficient than in the wild type. No interactions were detected between these residues in the contact map analysis, which suggests that the PD-SF coupling breaks. These results are supported by Cuello and coworkers where strong van der Waals interactions were observed between these residues in the pore-helix of the same subunit (Cuello et al., 2010).

It is noteworthy that the mutation of a residue implicated in one of the two pathways that defined the VSD-SF or the PD-SF couplings did not influence the other mechanism. However, the experimental studies showed that mutating a residue along one of the two paths (Perozo et al., 1993; Yang et al., 2007; Cuello et al., 2010; Cordero-Morales et al., 2011; Bassetto et al., 2021) determined the disruption only of that path which is sufficient to impair inactivation. This evidence suggests that the two communication pathways are not inter-changeable and both play a role in the C-type inactivation mechanism.

## 4 Discussion and Conclusion

In this study, a combined simulations/network theoretical approach was applied to identify dynamical pathways as the possible molecular basis of C-type inactivation in the K_*v*_1.2 channel. This dynamic analysis allowed us to identify two routes of motion propagation that occur during this transition. In particular, our results revealed that the constriction of the SF is coupled to the VSD displacement (i) through helix S5 and the P-Loop and (ii) through a direct connection with the PD.

In the first path, the inactivation starts from helix S4, which is the only part of the channel that responds to changes in the membrane potential. From here, the motion propagates to helix S5 of the neighboring subunit using two bridges in the middle helix, corresponding to residues V301 and I304, respectively. By mutating residues on the top of the helix, we noticed an impairment of inactivation, in agreement with the experimental results obtained in Shaker channels (Yang et al., 2007; Bassetto et al., 2021). It is noteworthy that this pathway encompasses residues L293 and F302, whose mutations into histidine and leucine, respectively, are known to modify the inactivation of K_*v*_1.2 leading to epileptic encephalopathy (Masnada et al., 2017; Pantazis et al., 2020).

Moreover, a second communication pathway was identified coupling the SF to the PD excluding the VSD. In this case, the starting point of the motion propagation is represented by the bending region on helix S6 whose movements, after the channel opening, are transferred to Y377 on the SF through the contact between I402 and T373. Interestingly, residue P405 lays on this path, and it is correlated to slight changes in the inactivation if mutated into a leucine: similar to L293H and F302L, mutation P405L has been shown to induce epilepsy (Syrbe et al., 2015).

Finally, in order to support our computational results, we mutated some residues implicated in the paths whose effects have been demonstrated to modify the inactivation in Shaker channels. The computed changes in the path lengths account for a delayed inactivation in the majority of cases while an acceleration occurs for W366F (see Table 2); these results yield microscopic insights into experimental results (Perozo et al., 1993; Yang et al., 2007; Cuello et al., 2010; Cordero-Morales et al., 2011; Bassetto et al., 2021), in particular on the allosteric mechanism of inactivation.

In summary, our work has unveiled the molecular determinants of C-type inactivation in the K_*v*_1.2 channel through a novel approach combining molecular dynamics simulations and network theoretical techniques. Two pathways were found coupling the VSD and the inner pore gate with the SF providing a tentative explanation of the inactivation mechanism. Interestingly, some pathological mutants shown to impair the inactivation lay on the pathways that we identified, strengthening our computational results.

## Data Availability

The datasets presented in this study can be found in online repositories. The names of the repository/repositories and accession number(s) can be found below: doi.org/10.5281/zenodo.5528762.
