# Supplementary material for: Exploring K v 1.2 Channel Inactivation Through MD Simulations and Network Analysis
Source: Front Mol Biosci. 2021 Dec 20;8:784276. doi: 10.3389/fmolb.2021.784276 (PMC8721119; doi:10.3389/fmolb.2021.784276)
Supplement: Supplementary file 1 [file Image2.pdf]

```
                                                     SF
hERG   586 LHNLGDQIGKPYNSSGLGGPSIKDKYVTALYFTFSSLTSVGFGNVSPNTN  635
                               |||.|    |.::...|:|:||:|::.|.|.
Kv1.2  359 --------------------PSIPD----AFWWAVVSMTTVGYGDMVPTTI  385
```
