# Supplementary material for: Exploring K v 1.2 Channel Inactivation Through MD Simulations and Network Analysis
Source: Front Mol Biosci. 2021 Dec 20;8:784276. doi: 10.3389/fmolb.2021.784276 (PMC8721119; doi:10.3389/fmolb.2021.784276)
Supplement: Supplementary file 2 [file DataSheet1.pdf]

Supplementary Material

1 SUPPLEMENTARY METHODS

In the network analysis, the key residues of the source regions were chosen arbitrarily in the middle of helices S4 and S6, at residues R303 and I402, respectively. Then, the radius of spheres centered at the key residues was chosen to be the most suitable (6 Å) to encompass the majority of the residues on those alpha helices defining the source regions. Several tests were done with radii values of 5, 6, 7, and 8 Å, which yield similar results. On the other hand, the sink region was chosen to be centered at Y377 (see the main text).

The Dijkstra's algorithm was used to compute the path of minimal distance between the Voltage Sensor Domain and the Pore Domain (the source regions) and the Selectivity Filter of the neighbouring subunit (the sink region).

In general, to apply Dijkstra's algorithm (Dijkstra, 1959) the nodes of the graph must be distributed in three sets:

- Set V is the set of nodes that have already been visited;
- Set F is the set of frontier nodes;
- Set S is the set of unknown nodes that includes all the other nodes of the graph.

In this algorithm it will be necessary to iteratively update two arrays:

- Array d is the array of minimal distances;
- Array u is the array of predecessors.

In the initialization stage, the source node x is put in the ensemble of frontier nodes while all distances in array d are set to infinity. All predecessors in array u are undefined. At each iteration of the algorithm, a node z in the frontier set F with minimal distance $d(z)$ is chosen and it is moved to the set V of visited nodes. Then, all unknown successors w of node z are moved from set S to set F. After that, the values $d(w)$ and $u(w)$ of all successors w of z are updated according to the following rules. If the old minimal distance of node w from the source node x is greater than the distance from the source node $d(z)$ of its predecessor z plus the length of the arc connecting z and w (p_{zw}), the distance $d(w)$ is updated to $d(z)$ + p_{zw}. If the distance $d(w)$ has been updated, the node z is the predecessor of w along a minimal path. Therefore the algorithm sets $u(w) = z$. These operations are repeated until either the destination node y is reached and added to the set of visited nodes V or the frontier set F has become empty. At the end, the algorithm will provide a minimal path connecting the source node x and the target node y along with the distance of this minimal path.

The Brandes algorithm (Brandes, 2001) was used to compute the betweenness of each residue implicated in the communication pathways. This algorithm allows to quantify the importance of each residue in the propagation of motion between the source and sink regions on the graph, computing the number of shortest paths that pass through each node. In particular, considering a general graph:

- σ_{st} is the number of shortest paths between nodes s and t;
- $\sigma_{st}(v)$ is the number of shortest paths between nodes s and t passing through v;

The betweenness of the v node is defined as:

$$B(v) = \sum_{s \neq v \neq t \in V} \frac{\sigma_{st}(v)}{\sigma_{st}} \tag{S1}$$

It can be computed following the steps:

- For every node v in V, set $B(v) = 0$;
- For each node s in V, find all the shortest paths between s and all other nodes. Store all these paths for each pair s, t;
- For each pair s, t, count the number of times v appears in the stored paths to give $\sigma_{st}(v)$ and divide by the total number of paths between s and t;
- $B(v)$ gives the final result.

The Brandes algorithm is a revised version of the Breadth-First Search (BFS) algorithm (Bundy and Wallen, 1984) that is used to identify the shortest paths between two nodes. For this reason, in order to understand its working principles is necessary to describe the main features of the BSF algorithm.

In the initial step, each node w is marked as unvisited by setting the distance between nodes s and w to infinity. Then, nodes that immediately precede w on a shortest path from s is set to the empty list and also the list of all shortest paths from s to w is set to empty list. At this point, the starting node s is chosen and it is put on the queue Q. The distance from s to itself is equal to zero. While Q is not empty, v is de-queued from Q. Now, for each node w such that is an edge in E from v to w, the distance from v to w to the distance from v to $v + 1$ is set and w is enqueued if the distance from v to w is infinity. On the contrary, if the distance from v to w is equal to the distance from v to $v + 1$, v is appended to the nodes that immediately precede w on a shortest path from s to the empty list. Finally, all paths by following nodes preceding node t back to s for each t are collected, storing the paths on the list of all shortest paths from s to w.

The Brandes algorithm makes use of a recursive step in the backward phase to allow direct calculation of the ratios for each v on the basis of its successor nodes on the shortest paths to every following t. For more details, see Ref. (Brandes, 2001).

2 SUPPLEMENTARY FIGURES

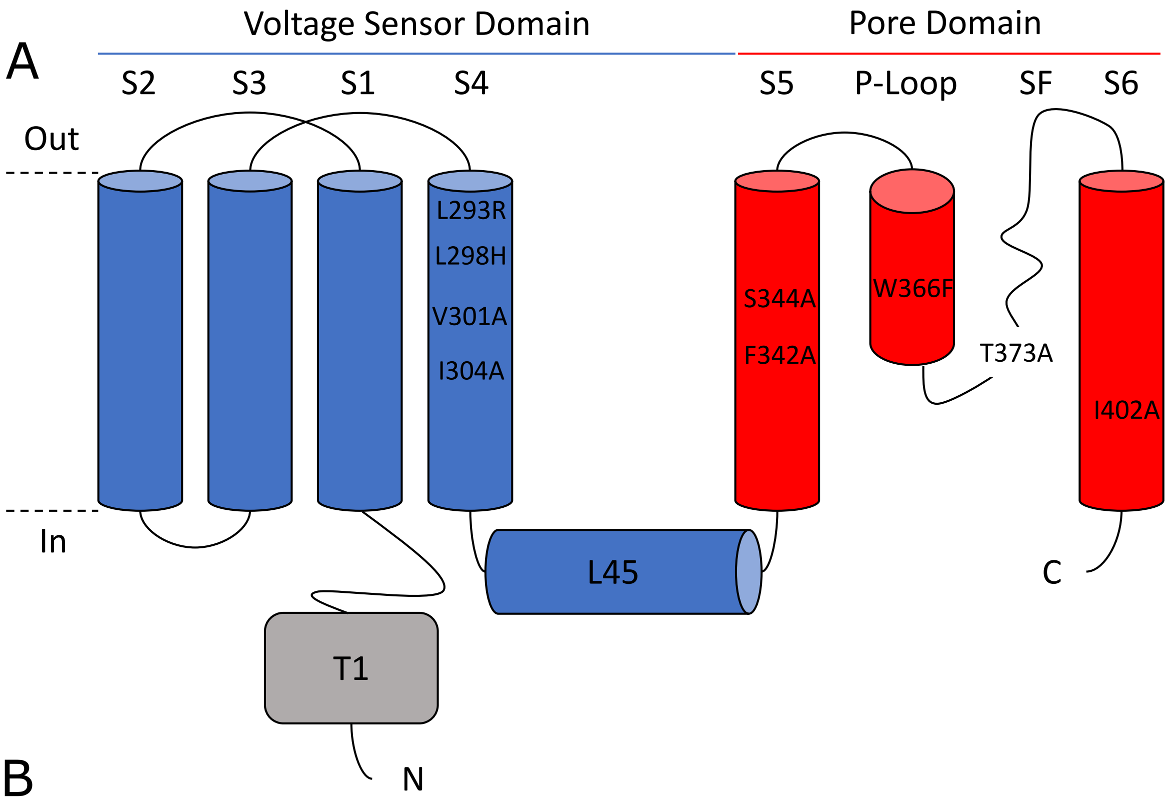

B

```
Shaker  339 NLPKAPVSPQD-KSSNQAMSLAILRVIRLVRVFRIFKLSRHSKGLQILGR    387
                    |:| :...|||||||||||||||||||||||||||||||||:
Kv1.2   278 --------PEDAQQGQQAMSLAILRVIRLVRVFRIFKLSRHSKGLQILGQ    319

Shaker  388 TLKASMRELGLLIFFLFIGVVLFSSAVYFAEAGSENSFFKSIPDAFWWAV    437
            ||||||||||||||||||||:||||||||||||....|.|.||||||||||
Kv1.2   320 TLKASMRELGLLIFFLFIGVILFSSAVYFAEADERESQFPSIPDAFWWAV    369

Shaker  438 VTMTTVGYGDMTPVGVWGKIVGSLCAIAGVLTIALPVPVIVSNFNYFYHR    487
            |:|||||||||.|..:.|||||||||||||||||||||||||||||||||
Kv1.2   370 VSMTTVGYGDMVPTTIGGKIVGSLCAIAGVLTIALPVPVIVSNFNYFYHR    419
```

Figure S1. Mutated residues implicated in the inactivation paths on the Kv1.2 structure **(A)** and on the sequence of the *Drosophila* Shaker channel (UniProt ID P08510) and the Kv1.2 *Shaker-like* human channel (UniProt ID P16389) after the sequence alignment **(B)** using the EMBOSS Needle tool (Li et al., 2015). For simplicity, in the sequence alignment only the VSD (helix S4 and L45), the P-Loop, the SF, and the PD (helices S5 and S6) are reported.

```
                                                 SF
hERG  586 LHNLGDQIGKPYNSSGLGGPSIKDKYVTALYFTFSSLTSVGFGNVSPNTN    635
                             |||.|    |.::...|:|:||:|::.|.|.
Kv1.2 359 -------------------PSIPD----AFWWAVVSMTTVGYGDMVPTTI    385
```

Figure S2. Sequence alignment of the hERG human channel (UniProt ID Q12809) and the Kv1.2 *Shaker-like* human channel (UniProt ID P16389) using the EMBOSS Needle tool (Li et al., 2015). The residues of the Selectivity Filters are highlighted in the green box.

REFERENCES

Brandes, U. (2001). A faster algorithm for betweenness centrality. *Journal of mathematical sociology* 25, 163–177

Bundy, A. and Wallen, L. (1984). Breadth-first search. In *Catalogue of artificial intelligence tools* (Springer). 13–13

Dijkstra, E. W. (1959). A note on two problems in connexion with graphs. *Numerische mathematik* 1, 269–271

Li, W., Cowley, A., Uludag, M., Gur, T., McWilliam, H., Squizzato, S., et al. (2015). The EMBL-EBI bioinformatics web and programmatic tools framework. *Nucleic acids research* 43, W580–W584
