# Supplementary material for: Exploring K v 1.2 Channel Inactivation Through MD Simulations and Network Analysis
Source: Front Mol Biosci. 2021 Dec 20;8:784276. doi: 10.3389/fmolb.2021.784276 (PMC8721119; doi:10.3389/fmolb.2021.784276)
Supplement: Supplementary file 3 [file Image1.pdf]

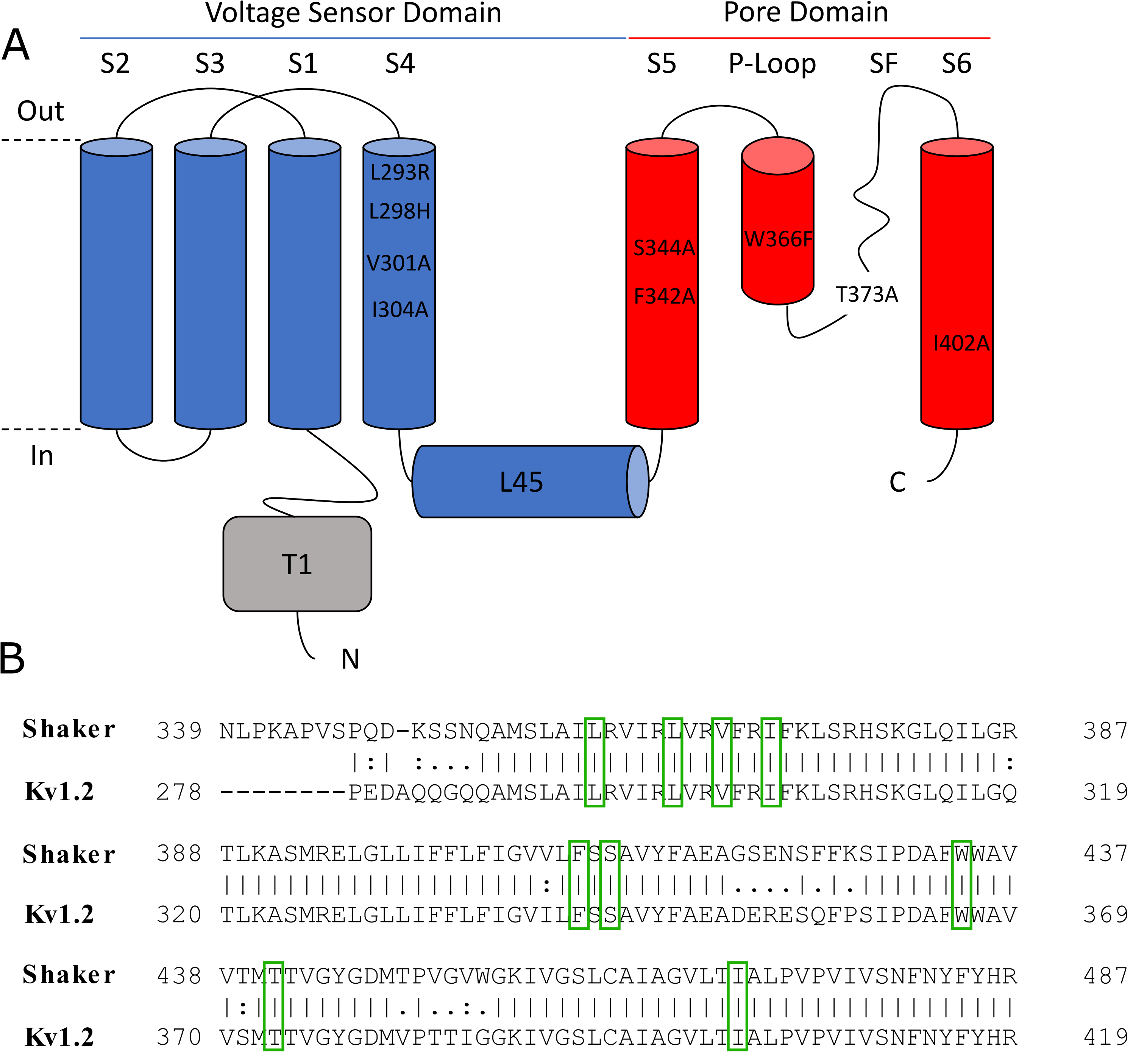
A
Voltage Sensor Domain
Pore Domain
S2
S3
S1
S4
S5
P-Loop
SF
S6
Out
In
L293R
L298H
V301A
I304A
S344A
F342A
W366F
T373A
I402A
L45
T1
N
C
B
Shaker 339 NLPKAPVSPQD-KSSNQAMSLAILRVIRLVRVFRIFKLSRHSKGLQILGR 387
Kv1.2 278 ---------PEDAQQGQQAMSLAILRVIRLVRVFRIFKLSRHSKGLQILGQ 319
Shaker 388 TLKASMRELGLLIFFLFIGVVLFSSAVYFAEAGSENSFFKSIPDAFWWAV 437
Kv1.2 320 TLKASMRELGLLIFFLFIGVILFSSAVYFAEADERESQFPSIPDAFWWAV 369
Shaker 438 VTMTTVGYGDMTPVGVWGKIVGSLCAIAGVLTIALPVPVIVSNFNYFYHR 487
Kv1.2 370 VSMTTVGYGDMVPTTIGGKIVGSLCAIAGVLTIALPVPVIVSNFNYFYHR 419
